# Swift thermal steering of domain walls in ferromagnetic MnBi stripes

**DOI:** 10.1038/srep24411

**Published:** 2016-04-14

**Authors:** Alexander Sukhov, Levan Chotorlishvili, Arthur Ernst, Xabier Zubizarreta, Sergey Ostanin, Ingrid Mertig, Eberhard K. U. Gross, Jamal Berakdar

**Affiliations:** 1 Institut für Physik, Martin-Luther-Universität, Halle-Wittenberg, D-06099 Halle/Saale, Germany; 2 Max Planck Institute of Microstructure Physics, D-06120 Halle/Saale, Germany

## Abstract

We predict a fast domain wall (DW) motion induced by a thermal gradient across a nanoscopic ferromagnetic stripe of MnBi. The driving mechanism is an exchange torque fueled by magnon accumulation at the DWs. Depending on the thickness of the sample, both hot-to-cold and cold-to-hot DW motion directions are possible. The finding unveils an energy efficient way to manipulate DWs as an essential element in magnetic information processing such as racetrack memory.

Domain walls (DWs), i.e. the cross border of regions with homogeneous but differently oriented magnetization play a central role in magnetism^1^. A particularly interesting suggestion is to exploit DWs for high density storage in a “racetrack” shift memory^2^,^3^. The shifting is brought about by passing a spin-polarized current that exerts a torque on the DWs^4^,^5^. While the proposal is technologically attractive it is hampered by large energy dissipation due to the high current densities needed. Recent advances resulted in an increase of the DW’s velocity at lower current densities^6^,^7^,^8^. It is however of interest to explore qualitatively new ways for controlling DWs. Here we show that magnonic current may serve as an efficient tool for the driving of DWs.

When a temperature gradient is applied to the system, it generates a magnon current and acts with a torque on the DWs (No voltage bias is applied. The drift motion of the charge carriers does not affect the magnetic configuration. We note, the DWs considered here are macroscopically large on the scale of the Fermi wave length^9^). Strong magnetocrystalline anisotropy impedes motion of DWs. Thus, the choice of material is essential. So far, only materials with a weak magnetocrystalline anisotropy received considerable attention. A particular example is a magnetic nanowire of permalloy (Ni_0.8_Fe_0.2_)^10^. Yttrium iron garnet (YIG) Y_3_Fe_2_(FeO_4_)_3_, Y_3_Fe_5_O_12_ is frequently used for thermal activation of spin currents (called spin Seebeck effect SSE)^11^,^12^,^13^,^14^ and has a slightly higher anisotropy (*K*_ul_(YIG) = −2.0 · 10^3^ J/m^3^ vs. *K*_ul_(permalloy) = −1.0 · 10^3^ J/m^3^)^15^.

Here we focus on manganese-bismuth compound MnBi, generally known as a hard ferromagnet^16^, where due to a strong magnetocrystalline anisotropy the ground state possesses an out-of-plane magnetization orientation. In addition, a remarkable behavior for the temperature dependence of the magnetic anisotropy of MnBi was observed: Experiments and first-principles calculations reveal that the out-of-plane magnetic anisotropy in MnBi increases at elevated temperatures^17^,^18^. As shown here, the notable temperature dependence of the anisotropy leads to a novel physical phenomena such as the acceleration of the domain wall motion. Increasing the applied temperature gradient in MnBi results in two distinct effects: It increases the magnonic spin current that drives the domain wall and creates gradient of the magnetocrystalline anisotropy. The width of a DW scales according to 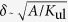. Here *A* is the exchange stiffness. A large magnetocrystalline anisotropy results in relatively narrow domain walls, i.e. a sharper noncollinear magnetic order and a larger exchange energy between the neighboring magnetic moments 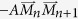. Hence, the energy landscape forces the motion of the DW to the area of a lower magnetocrystalline anisotropy and a lower exchange energy. Thus, the temperature dependence of the magnetic anisotropy in the hard ferromagnet MnBi generates a fundamentally new type of spin torque, which was not studied before. The magnetocrystalline anisotropy torque acts in addition to the applied thermal bias leading to a substantial enhancement of the DW’s velocity.

For a comprehensible study of the thermally activated DW motion all possible effects related to the applied thermal gradient should be addressed. An applied thermal bias generates a magnonic spin pumping current (a flux of magnons directed from the hot to the cold edge of the sample). On the path to the cold edge the magnonic current traverses the DW. Our calculations (see bellow) show that under certain conditions two different scenarios of the DW motion are realized: If the width of the DW is small enough (less than 7 nm, see Supplementary Information) the DW is transparent for thermal magnons and the magnons pass through the DW without a sizeable change of magnons’s momentum. Naturally, the spin pumping current does not exert a magnonic pressure on the DW’s surface, while the angular momentum is still transferred (the angular momentum is changed by 2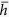 and the momentum of the magnon is conserved). A shortage of the angular momentum appears in the vicinity of the hot edge, while an extra angular momentum is accumulated at the cold edge of the sample (magnon accumulation effect, see^19^). In order to compensate for the imbalance in the distribution of the angular momentum, the DW propagates in the direction opposite to that of the magnon^20^,^21^. As for the direction of the thermally-induced DW’s motion, one should also consider the entropic torque. The free energy 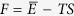 (here 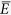 is the internal energy and *S* is the entropy) is minimized at elevated temperatures *T*. Thus, the entropic torque also provokes a motion of DW towards the hotter edge^22^,^23^,^24^,^25^,^26^. One may further argue that the DW is not thermally isolated and the heat flux associated with the magnonic current influences the entropy of the DW^27^. However, the influence of the entropic torque on the DW motion is an established trend with the DW moving towards the hot edge.

For a DW width exceeding 15 nm, the magnonic spin pumping current is totaly reflected by the DW. Therefore, in this case a second scenario is relevant: The magnons exert a sustainable pressure on the DW’s surface. If the magnonic pressure is strong enough, a thermally activated spin pumping current drags the DW to the cold edge. Our full numerical calculations evidence that indeed the direction of the DWs motion is system dependent. The microscopic theory^28^ predicts a strong magnonic recoil effect for the DW motion. The momentum of the DW depends on the tilt angle of its plane. If a magnon is reflected by the DW, the conservation of the total angular momentum induces a rotation of the DW. Thus, the magnon reflection leads to a motion of the DW in the direction of magnonic current^28^,^29^. The pressure exerted by the magnonic current on the DW reads: 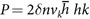. Here 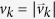, 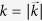 are the velocity and the wave vector of magnons, *δn* = *n*_*neq*_(*T*) − *n*_*eq*_(*T*) quantifies the magnon accumulation effect, i.e. the excess of the density of non-equilibrium magnons *n*_*neq*_(*T*) compared to the reference number of equilibrium magnons *n*_*eq*_(*T*) at the same temperature, but in the absence of the thermal gradient. Exerted pressure is proportional to the applied thermal bias. Our calculations (see Supplementary Information) are in line with the fact that magnons created in the hot area propagate toward the cold area. The left (right) side of the DW’s (see Fig. 1) is associated with the high (low) temperature of the applied thermal gradient. Therefore, the magnon accumulation is positive *δn* > 0 at the left side of the DWs and is negative *δn* < 0 at the right side exerting a magnonic pressure that drives the DWs. The direction of the motion of DW depends considerably on the ratio between the reflected and the transmitted magnons. The velocity of the DW can then be evaluated according to^30^


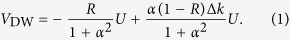


Here 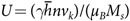, *α* is the phenomenological Gilbert damping, *γ* is the gyromagnetic ratio, *M*_*s*_ is the saturation magnetization, Δ is the width of the DW and *k* is the magnon’s wave vector, and 0 < *R* < 1 is the transmission coefficient. Driving DW via the spin waves excited by a microwave field shows a strong frequency dependence^20^. In contrast to the field-driven DW motion^31^, the thermally activated magnonic spin current is non-monochromatic^32^. Spectral characteristics of the magnons contributing to the magnonic accumulation are not yet fully settled. The magnonic current could be due to the difference between the magnon and the phonon temperatures^33^. Recent spatially resolved experiments carried out by Brillouin light scattering^34^ show a non-vanishing spin current for equal magnon and phonon temperatures. However, as shown in^35^, the thermally activated spin current is dictated by the magnon temperature profile rather than by the difference between the phonon and the magnon temperatures. In a recent experiment^36^ low frequency subthermal magnons were identified as a source for the spin current. These are magnons with a frequency lower than the internal cutoff frequency 
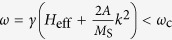
 (here *A* is the exchange stiffness and *H*_eff_ stands for the effective magnetic field). The internal cutoff frequency is not related to the structure of the DW but to the spectrum of the thermally excited magnons. The frequency spectrum of the thermally excited magnons is not monochromatic and covers a certain frequency domain. The internal cutoff frequency *ω*_*c*_ is the highest frequency of the magnons, which are excited thermally. Hence, the magnons with frequencies higher than the internal cutoff frequency *ω* > *ω*_*c*_ do not contribute to the thermally activated magnonic current^36^,^37^. We observed that magnons can either pass through the DW or are reflected off the DW depending on the geometry (thickness) of DW itself, while the spectral characteristics of the magnons (defined by thermal bias) are irrelevant. We find that in the case of a non-monochromatic, thermally activated magnonic current the ratio between the reflected and the transmitted magnons depends crucially on the characteristics of the DW itself, rather than on the cutoff frequency *ω*_c_. For the same magnonic current we detect DWs with different geometry moving in the opposite direction.

## Results

**Figure 2:**
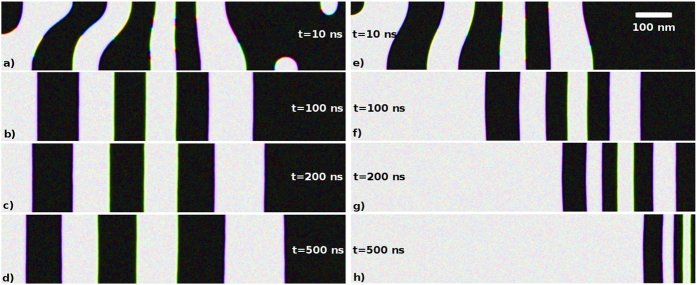
Top view of the magnetization configuration with no temperature gradient applied 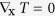 (left panel, (**a**–**d**)) and the magnetization configuration for both 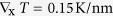 and *K*_ul_(*T*) = (9.69 · 10^3^ *T* − 1.12 · 10^6^) J/m^3^ (right panel, (**e**–**h**)) of a 1000 nm × 200 nm × 25 nm MnBi sample at *T*_*C*_ = 300 K and different time moments in the range [0:500] ns. The light color represents the magnetization pointing towards the reader and the dark color indicates the magnetization in the opposite direction both perpendicularly to the surface of the figure (cf. Fig. 1). The magnetization configuration is calculated including long-range interactions (the demagnetizing fields). The anisotropy strength is set according to first-principles calculations of ref. 18 fitted for the interval of [300:400] K (right panel, (**e**–**h**)). A thermal bias is applied to the sample such that *T*_*H*_ − *T*_*C*_ = 150 K. All simulations were performed with the initial state at *t* = 0 being always chosen randomly with regard to the magnetization orientation. Then, the magnetization of a given sample was relaxed up to 10 ns in the absence of an external thermal bias.

**Figure 3:**
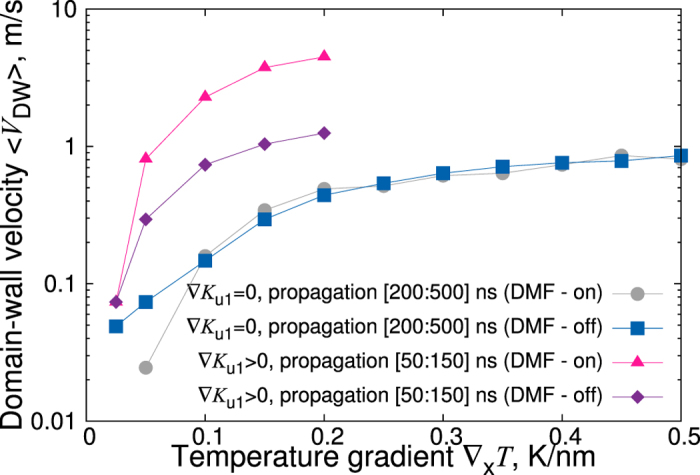
Dependence of the average domain-wall velocity on the temperature gradient 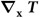 for a 1000 nm × 200 nm × 25 nm MnBi sample. The parameters are taken from Table 1 (Supplem. Information) and for *α* = 1.0. “DMF” stands for demagnetizing fields which are taken (or not) into account for the respective curves. Solid circles and squares represent the data for zero anisotropy gradient 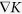. Triangles and diamonds show the situation with non-zero anisotropy gradient according to the first-principles calculations of ref. 18 fitted for the interval of [300:400] K as *K*_ul_(*T*) = (9.69 · 10^3^ *T* − 1.12 · 10^6^) J/m^3^. The increase of the DW’s velocity is a cooperative effect of the anisotropy gradient and the demagnetization fields. When the magnetocrystalline anisotropy gradient is applied the demagnetization field contributes to the formation of domains. We observed (not shown) that domains in this case are smaller and this enhances their mobility.

As shown in Fig. 2(a–d), a homogeneously heated sample does not lead to a directed domain wall motion in the sample, but rather a redistribution of domains on a long time scale is observed. Considering the time laps of [10:500] ns while a linear temperature gradient is applied and accounting also for the temperature dependence for the magnetocrystalline anisotropy^18^ (the anisotropy is stronger for higher temperatures), we observe (cf. Fig. 2(e–h)) that almost all domains reach the cold edge of the sample. Also the size of FM domains is reduced during their motion. The pressure exerted by the magnon accumulation effect modifies the shape of DWs. Figure 3 illustrates the domain wall motion. The averaged velocity of the domain wall 〈*V*_DW_〉 is plotted as a function of the linear temperature gradient. As expected, the velocity grows with elevating the temperature gradient, however it shows a slight saturation at higher gradients. The reason for that is the saturation of the magnonic current which was theoretically predicted in ref. 38. Surprisingly, the demagnetizing fields do not influence much the domain wall speed for a homogeneous anisotropy (Fig. 3, circles and squares). The inhomogeneity of the anisotropy induced by the temperature gradient increases the speed of domain wall motion by approximately one order of magnitude.

**Figure 4:**
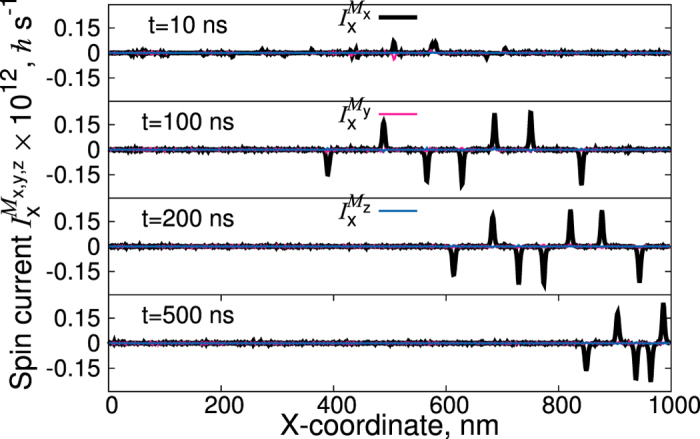
Time-resolved magnon spin currents corresponding to magnetic configurations shown in Fig. 2 (right panel) of a 1000 nm × 200 nm × 25 nm MnBi sample for the temperature gradient of 0.15 K/nm at different times in the range [0:500] ns. The left side of the sample is hotter, the right edge is always kept at room temperature. The magnetization configuration is calculated for *non-zero* demagnetizing fields. Anisotropy gradient is chosen according to first-principles calculations of ref. 18 fitted for the interval of [300:400] K in the form *K*_ul_(*T*) = (9.69 · 10^3^ *T* − 1.12 · 10^6^) J/m^3^.

**Figure 5:**
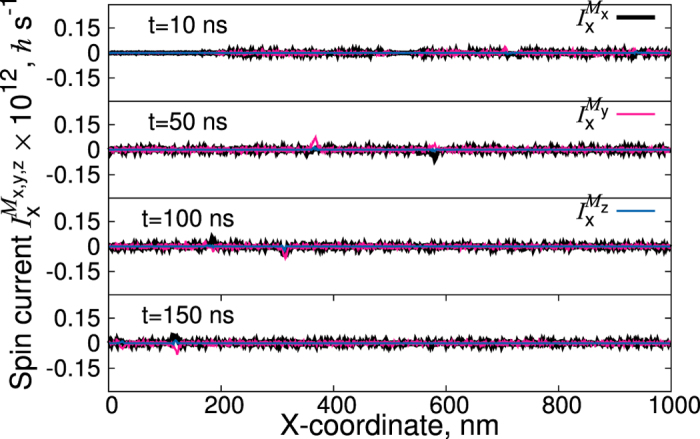
Time-resolved magnon spin currents of a 1000 nm × 200 nm × 1 nm MnBi sample for the temperature gradient of 0.15 K/nm at different times in the range [0:150] ns. The left side of the sample is hotter, the right edge is always kept at room temperature. The magnetization configuration is calculated for *non-zero* demagnetizing fields. The anisotropy is homogeneous, i.e. *K*_ul_ = 1.75 · 10^6^ J/m^3^.

Our general interpretation of the obtained DW dynamics relies on the definition of the magnon spin current given in ref. 35 by a recursive formula (Recurcive relation for the spin current: 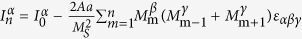, where *ε*_*αβγ*_ is the Levi-Civita antisymmetric tensor, *A* effective exchange stifness, *a* lattice constant, and *M*_*S*_ saturation magnetization. Greek indexes define the current components and the Latin ones denote sites). The x-component of the spin current 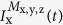 is calculated in a time-resolved manner. Figure 4 demonstrates clearly that most crucial for the magnon spin current is the presence of FM domain walls, where different components of the magnon-current tensor show pronounced peaks. The accumulation of the magnons at the domain walls leads to a torque acting on the domain walls, shifting them towards the colder edge. The situation might be different if the thickness of the sample is drastically reduced. In this case the magnonic spin current penetrates the DWs (Fig. 5) and the entire domain walls move towards the hotter electrode. However in this case the DWs move opposite to the anisotropy gradient. Therefore, the velocity of the DWs decreases.

## Discussion

We studied thermally activated motion of the DWs in the manganese-bismuth compound MnBi, generally known as a hard ferromagnet. We found that the unusual dependence of the magnetocrystalline anisotropy on the temperature (magnetocrystalline anisotropy in MnBi increases at elevated temperatures) is advantageous for a fast thermal steering of DWs. Sharp noncollinear magnetic orders formed under the strong magnetocrystalline anisotropy are energetically unfavorable. Therefore, the DWs slip to areas of small magnetocrystalline anisotropy. Thus, the gradient of the magnetocrystalline anisotropy acts as a macroscopic driving assisting the thermally activated motion of the DWs. The microscopic mechanism for the DWs motion is based on the magnonic spin current. The reflected magnonic spin current drags the DW to the cold edge while transmitted current forces the DW to the hot edge. We also observe a deformation of the shape of DWs due to the pressure exerted by the accumulation of magnons.

## Methods

To provide parameters for our model simulations we performed extensive first-principles studies of MnBi using a self-consistent Green function method within the framework of the density functional theory^39^. The ground state properties were calculated for the experimental lattice constant, while for the magnetic anisotropy energy (MAE) calculations the crystalline structure parameters were adopted from experiments, which studied the dependence of the MAE on the applied temperature^17^. First of all, we found that our simulations within the generalized gradient approximation provide an adequate description of MnBi. The main ground state properties, obtained within our calculations, are listed in Table 1, second column (Supplem. Information). Thus, in accordance with experiments, MnBi is a robust ferromagnet with Curie temperature of 680 K. The magnetic moments and exchange interaction parameters, estimated within the magnetic force theorem, provide the exchange stiffness close to the experimental values (cf. Table 1 (Supplem. Information)). The MAE calculated for *T* = 0 K and for higher temperatures is in a good agreement with the experimental results and other theoretical studies^17^,^18^. The influence of the conductance electrons is incorporated in the effective exchange stiffness^40^.

With the microscopic parameters delivered by experiments and first principles calculations we performed micromagnetic simulations for the magnetization dynamics on the basis of Landau-Lifshitz-Gilbert (LLG) equation, as implemented in the mumax3-micromagnetic simulation package^41^.

## Additional Information

**How to cite this article**: Sukhov, A. *et al*. Swift thermal steering of domain walls in ferromagnetic MnBi stripes. *Sci. Rep.* **6**, 24411; doi: 10.1038/srep24411 (2016).

## Supplementary Material

Supplementary Information

## Figures and Tables

**Figure 1:**
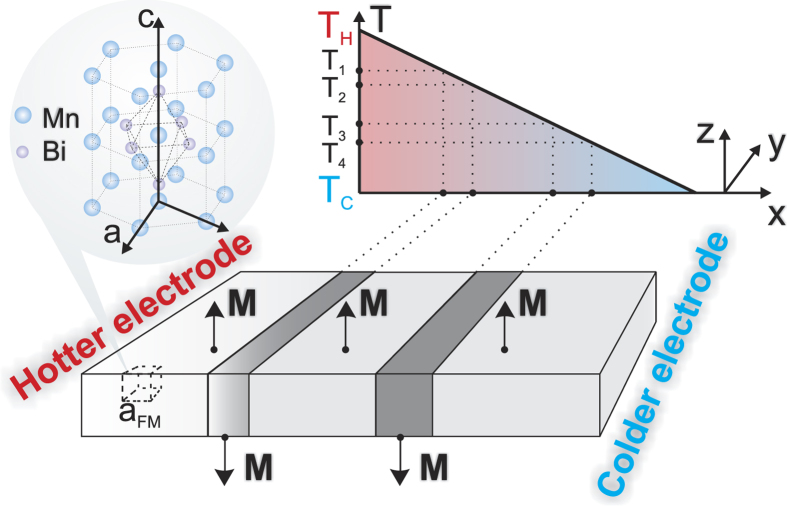
Schematics of the considered structure with a thermal bias applied along the x-axis. 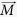
 indicates the direction of the magnetization within a domain, *a*_FM_ = 1 nm represents the coarse-grained FM cell including the average over several MnBi unit cells, whose crystallographic *c*-axis coincides with the z-axis.
