# Supplementary material for: Swift thermal steering of domain walls in ferromagnetic MnBi stripes
Source: Sci Rep. 2016 Apr 14;6:24411. doi: 10.1038/srep24411 (PMC4830975; doi:10.1038/srep24411)
Supplement: Supplementary Information [file srep24411-s1.pdf]

Supplementary Information: Swift thermal steering of domain walls in ferromagnetic MnBi stripes

Alexander Sukhov[1], Levan Chotorlishvili[1], Arthur Ernst[2], Xabier Zubizarreta[2], Sergey Ostanin[2], Ingrid Mertig[1,2], Eberhard K. U. Gross[2], and Jamal Berakdar[1,*]

[1]Institut für Physik, Martin-Luther-Universität, Halle-Wittenberg, D-06099 Halle/Saale, Germany

[2]Max Planck Institute of Microstructure Physics, D-06120 Halle/Saale, Germany

[*]Jamal.Berakdar@physik.uni-halle.de

ABSTRACT

Methods

Table 1 gives an overview over the main parameters that are relevant for the magnetic MnBi-alloy. In the present calculations the parameters termed as "experimental values" (third column) were employed. One of the main parameter that is important for the magnetic configuration is the ferromagnetic (FM) cell a_{FM}. It should be chosen such that detailed magnetic structures such as FM domains can be resolved. It is known[1] that the width of FM domain walls scales according to $\delta \sim \sqrt{A/K_{\mathrm{u1}}}$, where A is the exchange stiffness. Since the magneto crystalline anisotropy K_{u1} in MnBi is very high, it results in a relatively narrow domain walls with widths around $2-6$ nm. To resolve fine structures within the domain walls we deemed, upon tests, that $a_{\mathrm{FM}} = 1$ nm as appropriate. In the *ab-initio*-calculations we obtained the value for the saturation magnetization (calculated as $M_{\mathrm{S}} = \frac{\mu_S}{V_0}$, where $V_0 = 137.48$ Å^3 is a volume of the unit cell and $\mu_{\mathrm{S}}(T=0\mathrm{K}) = 8.4\mu_B$ or $\mu_{\mathrm{S}}(T=300\mathrm{K}) = 7.33\mu_B$ for MnBi) and the exchange stiffness constant (alculated based on the ratio $A = \frac{DM_{\mathrm{S}}}{2g\mu_{\mathrm{B}}}$ (in MnBi $D = 670$ meV Å^2), which is derived in Ref.[5]) shown in Table 1. For comparison, analogous values for the exchange stiffness (calculated from the ratio for the FM domain wall thickness $\delta_{\mathrm{FM}} = \sqrt{A/K_{\mathrm{u1}}}$ based on values from Ref.[4] at room temperature), the Gilbert damping (calculated based on the ratio $\alpha = \frac{\sqrt{3}}{2}\frac{\gamma}{\omega}\Delta B_{\mathrm{pp}}$, which is approximated for the half-height of the width of the FMR spectrum curve at $T = 130$ K. The values of $\Delta B_{\mathrm{pp}} \approx 0.05$ T, $\omega/(2\pi) = 9$ GHz are taken from Ref.[6] Gyromagnetic ratio is $\gamma = 1.76 \cdot 10^{11}$ (Ts)$^{-1}$) and the FM cell size (should be of the order of 2.5 nm, which is min. δ_{FM} (T=300 K) based on values of Ref.[4]) are also listed in Table 1.

The size of the simulated MnBi sample has the dimension of 1000 nm along the x-axis, 200 nm along the y-axis, and a thickness of 25 nm (Sections I, II and III). It required 1.2 GB of the GPU-memory while including demagnetizing fields. To

examine the dynamics of domain walls we focused on an stripe sample with the temperature gradient being along the stripe (chosen as the x-direction). The value $25 \div 30$ nm for the sample thickness was taken in accordance with the minimal known thickness where a magnetic pattern was still recognizable.[3]

In order to initiate FM domains, we proceed in a natural way by starting from a random magnetic configuration at zero magnetic field and at room temperature ($T = 300$ K). To achieve convergence to a well-defined magnetic configuration a relatively large damping was chosen which resulted in a convergence to the ground state on the time scale of several to ten nanoseconds (simulation time step was set to $5 \cdot 10^{-13}$ s). After reaching the ground state which is a magnetic pattern with several FM domain walls (typically after 10 ns), the temperature gradient is applied, where the hotter end is the left part of the sample, whereas the right end is always kept at room temperature.

The magnetic configurations with time are obtained from the solution of the Landau-Lifshitz-Gilbert (LLG) equation within the micromagnetic framework by means of `mumax3`-software.[7]

DESCRIPTION	*ab-initio*	EXPERIMENT
Magnetization M_S, [A/m]	$5.6 \cdot 10^5$ ($T = 0$ K)	$7.42 \cdot 10^5$ (T=0 K)[4]
	$4.9 \cdot 10^5$ ($T = 300$ K)	$6.40 \cdot 10^5$ (T=300 K)[4]
Exch. stiffness A, [J/m]	$1.43 \cdot 10^{-11}$	$6.25 \cdot 10^{-12}$
Anis. strength $K_{u1}(T)$, [J/m^3]	$-\,0.44 \cdot 10^6 (T = 0\,\mathrm{K})$[2]	
	$1.4 \cdot 10^6 (T = 300\,\mathrm{K})$[2]	$1.75 \cdot 10^6 (T = 300\,\mathrm{K})$[4]
Anisotropy type	uniaxial	uniaxial
Gilbert damping α	-	0.0054 (T=130 K)
Curie-Temperature T_C, [K]	680	775 (Ref.[4])
FM cell size a_{FM}, [nm]	-	2.5

Table 1. Parameters relevant for MnBi-alloy.

Domain-wall motion

Fig. 1 provides additional illustration of the DW motion under thermal bias.

Domain-wall thickness

In Figs. 2, 3 we demonstrate the trend in the thermally assisted DW motion with the DW thickness.

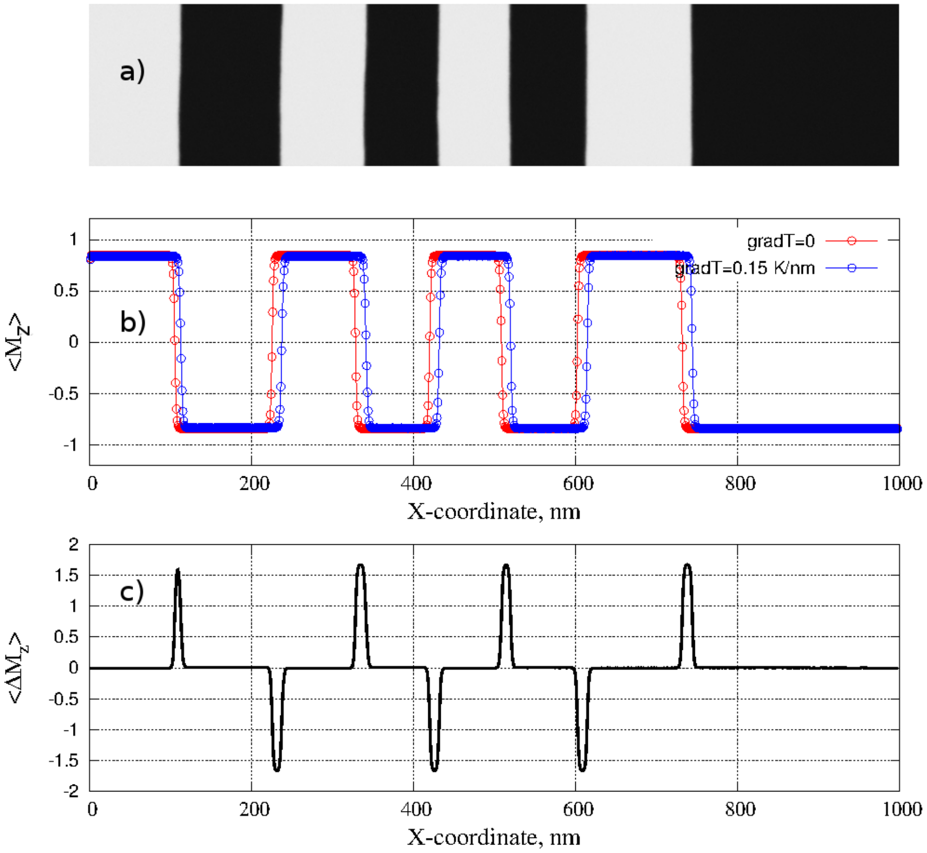

Figure 1. (a) Top view of the magnetization configuration (black color indicates the magnetization direction ”down”, white color implies the magnetization direction ”up”) of a 1000nm×200nm×25nm MnBi sample for the temperature gradient of 0.15 K/nm at time 100 ns. The spin accumulation (c) is calculated according to the expression $< \Delta M^n_z > = < M^n_z > |_{\bigtriangledown T \neq 0} - < M^n_z > |_{\bigtriangledown T = 0}$ for a 1000nm×200nm×25nm MnBi sample, where $< M^n_z > |_{\bigtriangledown T=0}$ (b) stands for the data with no temperature gradient at time $t = 100$ ns and $< M^n_z > |_{\bigtriangledown T \neq 0}$ (b) is for the data with a temperature gradient 0.15 K/nm at time $t = 100$ ns. (c) A positive spin accumulation corresponds always to the left side of domains, negative values of the spin accumulation are associated with the right side of the corresponding domains.

References

1. Coey, J. M. D. *Magnetism and Magnetis Materials* (Cambridge, 2010).

2. Antropov, V. P., Antonov, V. N., Bekenov, L. V., Kutepov, A. & Kotliar, G. Magnetic anisotropic effects and electronic correlations in MnBi ferromagnet, *Phys. Rev. B* **90**, 054404 (2014).

3. Dekker, P. *Magnetization reversal processes in thin MnBi films* (Dissertation, bibliothek TU Delft, 1974).

4. Guo, X., Chen, X., Altounian, Z., & Ström-Olsen, J. O. Magnetic properties of MnBi prepared by rapid solidification. *Phys. Rev. B* **46**, 14578 (1992).

5. Hamrle, J. et al. Determination of exchange constants of Heusler compounds by Brillouin light scattering spectroscopy: application to Co_2MnSi. *J. Phys. D: Appl. Phys.* **42**, 084005 (2009).

6. Chen, D., Gondo, Y. Temperature Dependence of the Magneto-Optic Effect and Resonance Phenomena in Oriented MnBi Films. *J. Appl. Phys.* **35**, 1024 (1964).

7. Vansteenkiste, A. et al. The design and verifcation of MuMax3. *AIP Adv.* **4**, 107133 (2014).

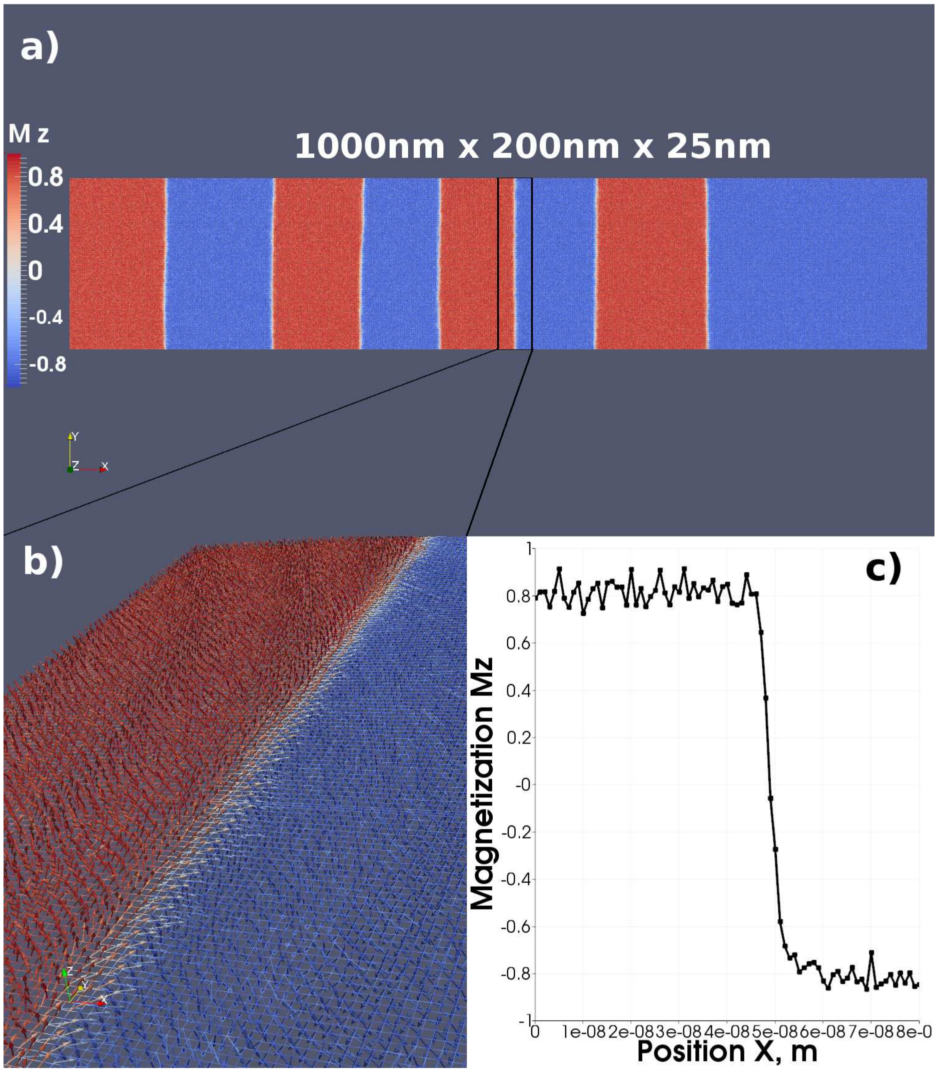

Figure 2. (a) Top view of the magnetization configuration (blue color indicates the magnetization direction ”down”, red color implies the magnetization direction ”up”) of a 1000nm$\times$200nm$\times$25nm MnBi sample for the temperature gradient of 0.15 K/nm at time 100 ns ($\nabla K_{u1} = 0$, DMF - on). (b) stands for the magnetization configuration frame between $[470:570]$ nm (top layer only). (c) indicates the averaged magnetization profile along a line drawn parallel to the x-direction and connecting edges of the frame $[470:570]$ nm for the top layer. The resulting domain-wall thickness is $d_{\mathrm{DW}} \approx 15$ nm.

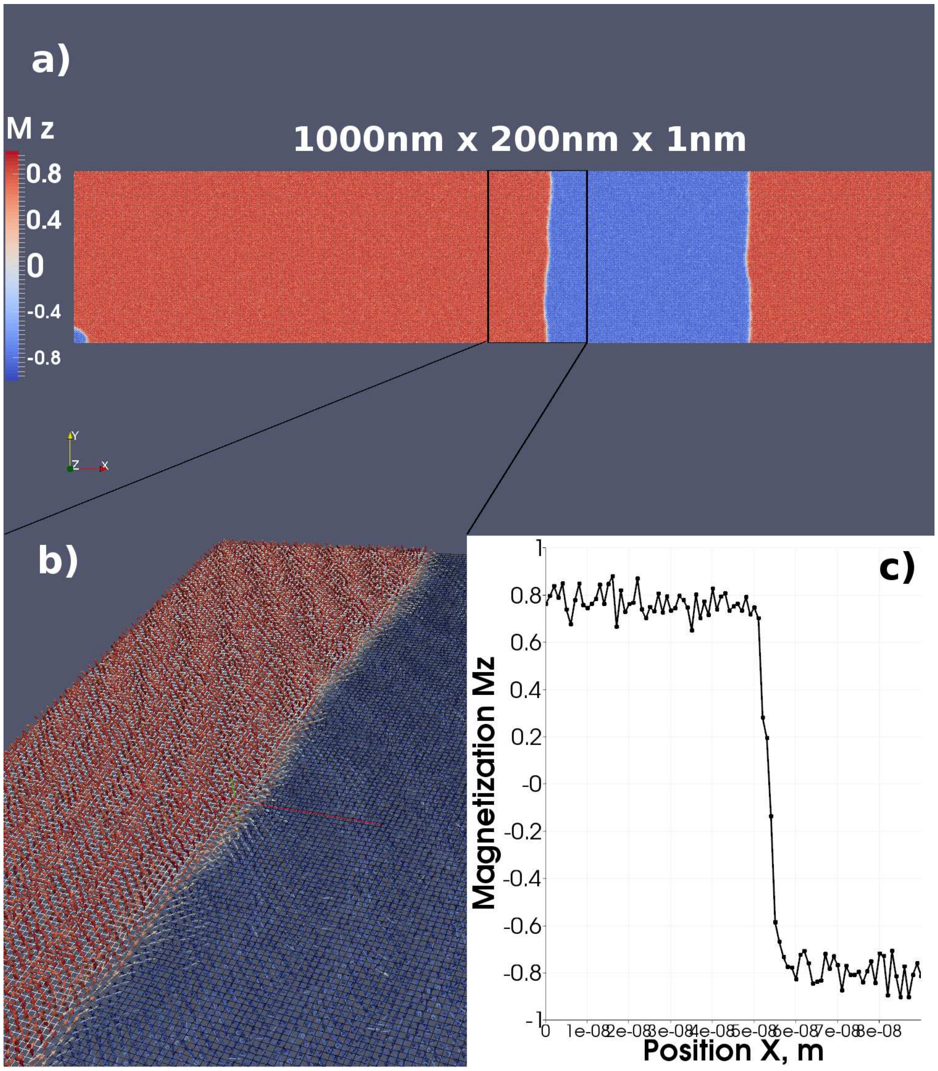

Figure 3. Same as in Fig. 2 but (a) for the temperature gradient of 0.15 K/nm at time 20 ns ($\nabla K_{\mathrm{u1}} = 0$, DMF - on). (b) the magnetization configuration frame is between $[500:600]$ nm. (c) the averaged magnetization profile for the frame $[500:600]$ nm. The resulting domain-wall thickness is $d_{\mathrm{DW}} \approx 7$ nm.
